# Identification and Characterization of Small RNAs in the Hyperthermophilic Archaeon *Sulfolobus solfataricus*

**DOI:** 10.1371/journal.pone.0035306

**Published:** 2012-04-13

**Authors:** Ning Xu, Yan Li, Ying-Tao Zhao, Li Guo, Yuan-Yuan Fang, Jian-Hua Zhao, Xiu-Jie Wang, Li Huang, Hui-Shan Guo

**Affiliations:** 1 State Key Laboratory of Plant Genomics and National Center for Plant Gene Research, Institute of Microbiology, Chinese Academy of Sciences, Beijing, China; 2 State Key Laboratory of Plant Genomics, National Center for Plant Gene Research, Institute of Genetics and Developmental Biology, Chinese Academy of Sciences, Beijing, China; 3 State Key Laboratory of Microbial Resources, Institute of Microbiology, Chinese Academy of Sciences, Beijing, China; 4 Graduate University of Chinese Academy of Sciences, Beijing, China; Duke University, United States of America

## Abstract

The term RNA silencing (RNA interference, RNAi) describes a set of mechanisms that regulate gene expression in eukaryotes. Small interfering RNAs (siRNA) and microRNAs (miRNAs) are two major types of RNAi-associated small RNAs (smRNAs) found in most eukaryotic organisms. Despite the presence of a plethora of non-coding RNAs longer than 50-nucleotide (nt) in length in various species of Archaea, little is known about smRNAs in archaea that resemble the 20–24-nt long smRNAs found in eukaryotes, which have been implicated in the post-transcriptional control of gene expression. Here, we report the finding of a large number of smRNAs approximatelly 20-nt in length, including phased smRNAs and potential miRNAs, from the hyperthermophilic archaeon *Sulfolobus solfataricus* p2 (Ssp2) based on deep sequencing. The expression of some of the miRNA candidates in Ssp2 was confirmed. Consistent with the Ssp2 hyperthermophilic properties, we found that higher temperatures more efficiently induced the production of the miRNA candidates in an *in vitro* system using the putative foldback precursor transcripts incubated with Ssp2 extract. Although we initially predicted putative target genes of some miRNA candidates, further analysis mapped the cleavage sites downstream of the miRNA candidate complementary regions, similar to those involved in plant miRNA-mediated *TAS* transcript cleavage. We also identified smRNAs from clustered, regularly interspaced, short palindromic repeat (CRISPR) loci, which play important roles in prokaryotic microbial defense systems. Archaea represent a unique life form next to Bacteria and Eukarya, and our results may provide a useful resource for further in-depth study on the regulation and evolution of smRNAs in this special organism.

## Introduction

RNA silencing (RNA interference, RNAi) refers to related homology-dependent gene silencing mechanisms in eukaryotic organisms that regulate gene expression, cell viability and stress/defense response, as well as many other processes. RNA silencing is guided by small RNAs (smRNAs) such as microRNAs (miRNAs) and small interfering RNAs (siRNAs) [1], [2], [3], [4], [5], [6]. In plants and animals, 20–24-nt miRNAs are generated from foldback precursor transcripts cleaved by Dicer (DCR) or Dicer-like (DCL) proteins, which can then directly cleave and translationally repress partially complementary target mRNA transcripts through conserved Argonaute (AGO) proteins that contain PAZ (oligonucleotide binding) and Piwi (active or inactivated nuclease) domains [7], [8], [9], [10]. The production of siRNAs is always triggered by long double-stranded RNAs formed between two overlapping antisense RNAs or long RNA transcripts with inverted complementarity [11], [12]. In plants, certain miRNAs indirectly regulate developmental processes by initiating the production of primary *TRANS-ACTING* RNA (*TAS*)-derived siRNAs (trans-acting siRNAs, tasiRNAs). The miRNA targeting site upstream of the tasiRNA-producing region is necessary and sufficient for triggering the formation of tasiRNA [13], [14], [15], [16], [17]. Because tasiRNAs are processed sequentially from *TAS*-transcript-derived dsRNAs, the cloned tasiRNAs often exhibit in 21-nt increments relative to the cleavage site on both strands (named phased smRNAs) [18]. miRNAs and siRNAs, including phased siRNAs, reminiscent of plant tasiRNAs have been reported in the unicellular alga *Chlamydomonas reinhardtii* [19], [20].

An RNAi-like defense system in prokaryotes (pRNAi) has recently been discovered [21], [22], [23], [24], [25]. The hallmark of the pRNAi system is the clustered regularly interspaced short palindromic repeats (CRISPR) locus, which is found in 90% of archaeal genomes and 40% of bacterial genomes [24], [26], [27], [28], [29], [30]. CRISPR/Cas (CRISPR-associated protein) constitutes an adaptive RNA-mediated defense system that targets invading phages or plasmids. A key event in CRISPR activation is the maturation of the active crRNAs (CRISPR-derived short guide RNAs) from the CRISPR precursor transcript (pre-crRNA) [31], [32]. In bacteria, trans-encoded small RNAs (tracrRNAs) transcribed upstream of the opposite strand of a CRISPR gene were recently found to participate in the maturation of crRNAs in the human pathogen *Streptococcus pyogenes* [33]. In the archaeon *Pyrococcus furiosus*, crRNAs that silence foreign nucleic acids in a sequence-specific manner by a CRISPR/Cas effector complex have also been identified [24], [25], [34]. In the archaeon *Sulfolobus solfataricus*, two types of CRISPR/Cas systems have been described. A type I-A complex, similar to *Escherichia coli* encodes type I-E CAS complex, is composed of several conserved subunits. The crystallographic structures of two protein subunits, Csa2 and Csa3, from *S.solfataricus* p2 (Ssp2) have been elucidated [35], [36]. A second type III-B system is composed of seven CAS protein subunits (Cmr1–7). The three-dimensional architectures of the full CMR complex and the subcomplex of Cmr2/Cmr3/Cmr7 have also been elucidated recently [37]. Archaea represent a unique life form next to Bacteria and Eukarya [38]. Despite the similar CRISPR/Cas immunity and their morphological similarity to bacteria [39], Archaea resemble eukaryotes in terms of their genetic mechanisms and some metabolic processes [40], [41], [42], [43]. A recent study [44] using a combination of whole transcript sequencing and strand-sensitive 5′-end determination discovered 310 expressed non-coding RNAs (ncRNAs) in the archaeon *S. solfataricus* p2 (Ssp2) with extensive expression of overlapping *cis*-antisense transcripts, suggesting that antisense-based mechanisms may also be widely used in Archaea to regulate gene expression in a manner similar to eukaryotes [44]. This archaeal transcriptome data also include small non-coding RNAs associated with L7Ae, a component of the large subunit of the ribosome, and small nucleolar RNA (snoRNA)-like RNAs of 50–60-nt in length, which were identified previously [45], [46], [47], [48]. snoRNAs direct ribosomal RNA (rRNA) processing and modification as well as function as ribonucleoprotein complexes in eukaryotes and archaea, suggesting that Archaea and Eukarya share a common ancestor that predates the evolution of a morphologically distinct nucleolus [45]. Recent studies have provided evidence for the coexistence of eukaryotic smRNA biogenesis and functionally related protein homologs in Archaea [49], [50], suggesting the existence of RNA-silencing pathways in Archaea similar to those in eukaryotes. However, there has been no report about archaeal smRNAs resembling that of eukaryotes 21–25-nt in length, which have been implicated in the post-transcriptional control of gene expression.

Here, we show that the hyperthermophilic archaeon *S solfataricus* p2 (Ssp2) contains both siRNAs and potential miRNAs, including phased smRNA and CRISPR-related smRNAs. We found that higher temperatures increase the production of miRNA candidates in an *in vitro* system. 5′RACE analysis of putative targets of miRNA candidates mapped the cleavage sites from several to hundreds of basepairs downstream of the aligned miRNA candidate regions, which is reminiscent of miRNA-dependent triggering of *TASs* transcript cleavage in plants. Our findings suggest that the siRNA/miRNA-related regulatory pathway may be an ancient mechanism of gene regulation that evolved prior to the emergence of eukaryotic cells.

## Results

### Deep sequencing analysis of *Sulfolobus solfataricus* smRNAs

**Figure 1:**
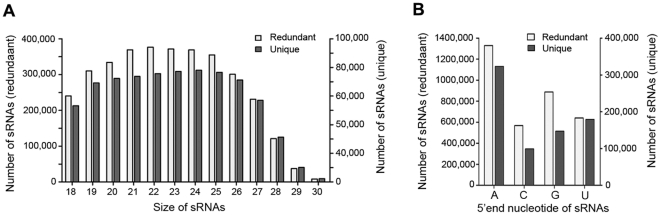
*S. solfataricus* smRNAs. (A) Size distribution of *S. solfataricus* p2 smRNAs. (B) Sequence composition of the 5′ end of the smRNAs. Redundant and unique smRNA reads are represented as white and gray bars, respectively.

A number of small non-coding RNAs longer than 50-nt in length in Archaea have been identified [45], [46], [47], [48]. To examine whether archaea encode smRNAs approximately 20-nt in length, similar to those identified in eukaryotes, total RNA was extracted from the hyperthermophilic archaeon *S. solfataricus* p2 (Ssp2) strain growing at the preferred temperature of 80°C. RNA fractions with sizes between 18 and 30 nucleotides based on PAGE analysis were collected and cloned. A total of 5,252,738 reads were obtained by deep sequencing. After removing unmatched nucleotides at either end of the smRNA reads, 3,424,144 sequences had at least one perfect match in the Ssp2 genome. Most sequences fell into the 18–29-nt range (Fig. 1A), however, there was no predominant peak in the 21–24-nt size class, as is found in eukaryotic smRNAs [51], [52], [53]. The smRNAs with perfect genomic matches represented 747,989 unique sequences. Of these, 536,535 smRNAs were cloned only once, indicating that the smRNA population in Ssp2 is complex. Unlike smRNAs in eukaryotic organisms that display a bias towards uridine (U) at the 5′ end [20], [54], [55], we observed a high percentage of adenosine (A) at the 5′ end in both redundant and unique smRNA sequences of Ssp2 (Fig. 1B).

**Figure 2:**
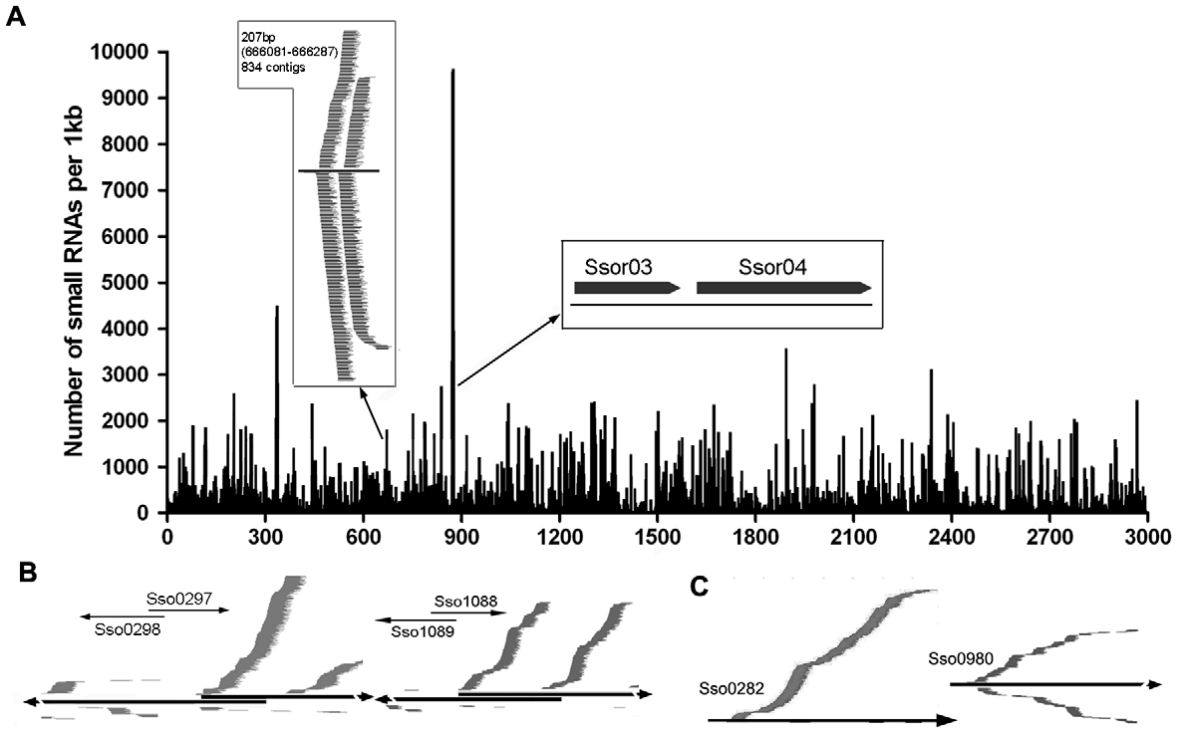
A catalog of *S. solfataricus* p2 smRNAs. (A) Genome-wide density analysis of cloned smRNAs (unique data set) in the Ssp2 genome. The number of smRNAs with perfect matches in either direct or complementary strand within each 1-kb window is plotted. A smRNA production hot spot that covered the 16S (Ssor03) and 23S (Ssor04) rDNAs (two black bars in the horizontal oblong insert) is shown. A smRNA-rich region (containing 834 smRNAs within a 207-bp sequence) corresponding to both strands that matched to intergenic regions is shown (vertical oblong insert). (B) SmRNAs are enriched in or on the border of the overlapping regions of neighboring genes. (e.g., protein IDs Sso0298 and Sso0297, Sso1088 and Sso1089). (C) smRNAs phased relative to each other at two representative loci (e.g. protein IDs Sso0282, Sso0980). The short thin lines *above* the black lines represent smRNAs derived from the sense strand, and those *below* the black lines represent smRNAs from the antisense strand.

The smRNA-generating regions include intergenic regions, annotated protein-coding genes, repetitive sequences and regions producing noncoding RNAs (rRNAs and tRNAs), as well as snoRNAs (Table 1). We summed the number of unique smRNAs in a 1-kb sliding window and plotted it against the whole genome of Ssp2. We observed one hot spot for smRNA production (Fig. 2A) that included 9630 smRNAs within a 1-kb sequence and matched to two rRNAs (Fig. 2A). Many other smRNA-rich regions matched to intergenic regions, for instance, 834 smRNAs mapped to a 207-bp sequence, in both strands (Fig. 2A). In addition, many smRNAs were found to be enriched in or on the border of the overlapping regions of neighboring genes (e.g., protein IDs Sso0298 and Sso0297, Sso1088 and Sso1089) (Fig. 2B), a feature shared with siRNAs derived from the widespread natural *cis*-antisense transcripts (nat-siRNAs) in eukaryotes [11], [12], [56]. This is also consistent with the recent discovery of a large number of noncoding RNAs in Ssp2 with extensive expression of overlapping *cis*-antisense transcripts at a level unprecedented in any bacteria or archaea but common in eukaryotes [44].

**Table 1:** *S. solfataricus* sRNAs matching different categories of sequences.

Categories	Redundant	Unique
Genome	3,424,144	747,989
mRNA	1,437,543	614,753
rRNA	238,386	22,772
tRNA	94,791	11,564
snRNA	158	138
snoRNA	426	147
repeats	177,941	29,031
other	1,473,523	69,393
miRNA candidate	1376	191

### Identification of phased smRNA clusters

The fact that many cloned smRNAs were found to be related to each other in smRNA production regions suggests the possibility of phased smRNAs like plant miRNA-mediated *TAS* transcript-derived tasiRNAs [14]. To test this idea, we used a sliding window analysis to look at the whole Ssp2 chromosome for smRNAs with lengths of 19, 20, 21, 22, 23, 24 and 25 nt.We obtained a total of 32,517 windows with 83 phased regions (Fig. 2C). These phased smRNA clusters ranged in size from 189 bp to 5039 bp. Among the 83 phased smRNA clusters, 25 were from protein-coding regions including transposon genes, hypothetical genes and characterized genes, 20 from intergenic regions, 2 from rRNAs and tRNAs regions and 36 from overlapping protein-coding regions and intergenic regions.

### CRISPR loci-related smRNAs

**Figure 3:**
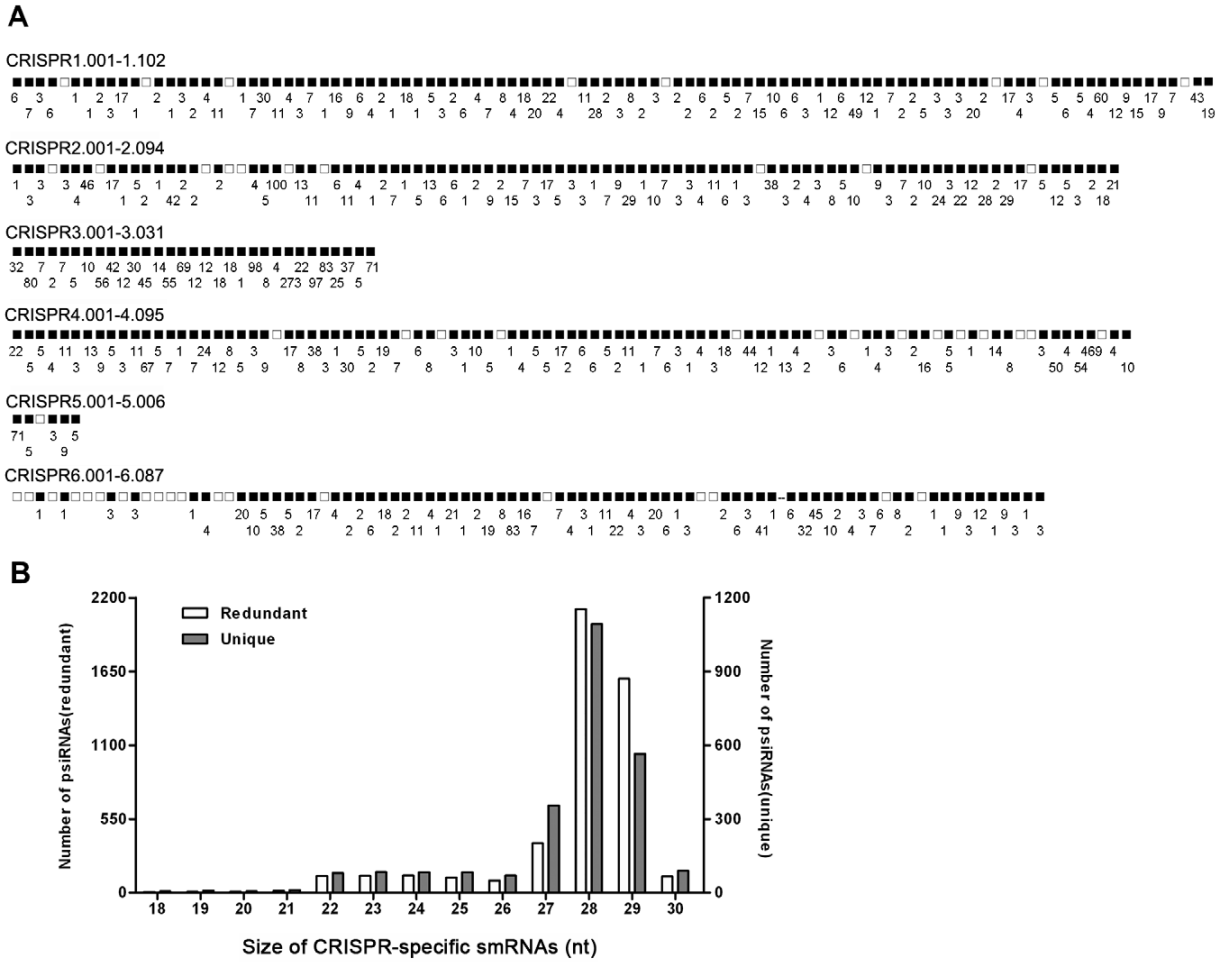
*S. solfataricus* p2 smRNAs matched to CRISPR loci. (A) Distribution of cloned smRNAs matched to the six Ssp2 CRISPR loci (The genome coordinates of each Ssp2 CRISPR locus are as follows: 1, 1233466–1239935; 2, 1254482–1260452; 3, 1297153–1299148; 4, 1305634–1311637; 5, 1744006–1744417; 6, 1809772–1811221). Each predicted Ssp2 CRISPR-derived short guide RNA (crRNA) locus is represented by a box, and the crRNA (box) numbers associated with each locus are indicated above. A shaded box indicates at least one smRNA clone matched to the locus in this work, and the number of smRNA reads is indicated below the shaded box. (B) Size distribution of CRISPR-specific smRNAs. Redundant and unique smRNA reads are represented as white and gray bars, respectively.

CRISPR/Cas loci are found in most archaeal genomes [57], [58]. *In vivo* CRISPR/Cas-mediated activity has been shown to target DNA in *S. solfataricus* [59]. However, both types I and III CAS complexes of *S. solfataricus* are also shown to contain ribonuclease activity and process pre-crRNA [36], [37]. In our smRNA library, smRNAs corresponding to CRISPR loci were also identified. A total of 177,941 contigs matched perfectly within the six CRISPR loci (Fig. 3A), and the length of most frequently cloned of the CRISPR-specific smRNAs was between 27 and 29 nt (Fig. 3B). Because the most abundant crRNAs in *P. furiosus* are 39–45 nt [24]. Moreover, crRNA is also targeted by the *S. solfataricus* type III CAS complex (SsoCMR) for cleavage in a sequence-dependent manner [37]. Therefore, we cannot rule out the possibility that these CRISPR-specific smRNAs are CMR-mediated cleavage products of Ssp2 crRNAs.

### Identification of potential miRNAs

**Figure 4:**
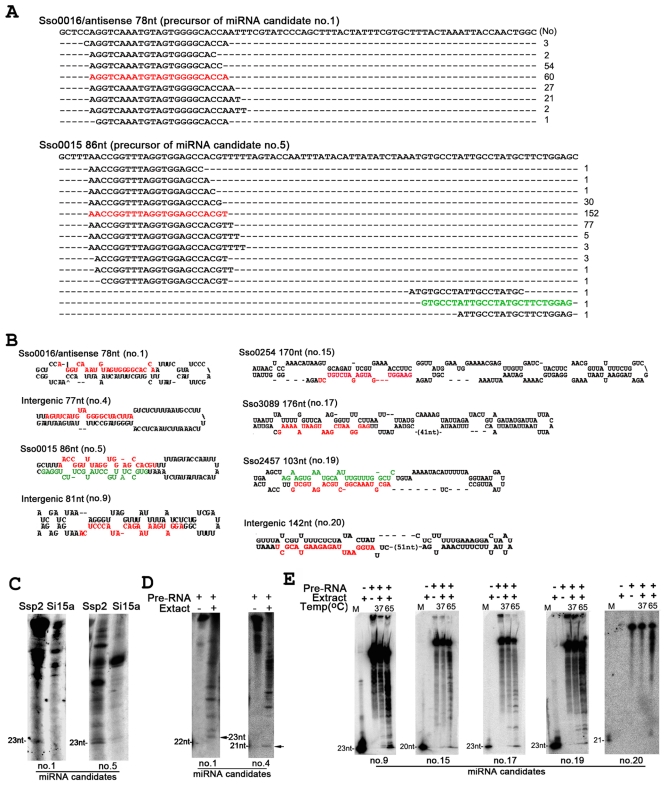
Representative miRNA candidates and their expression analysis in *S. solfataricus* p2. (A) Centered distribution of smRNAs around miRNA candidate no. 1 and 5. The sequences corresponding to the mature miRNA candidates (the most frequently cloned sequence in a miRNA candidate family) are shown in red and star strand (*) in green. (B) The predicted stemloop structures of selected miRNA candidate precursors (all putative precursor structures and genome IDs are shown in Supplementary Fig. S1). Putative precursor sequences were folded using the mfold (v3.2) program. (C) Expression profile analysis of the two most frequently cloned miRNA candidates (no. 1 and 5) with ^32^P-labelled oligodeoxynucleotide probes. The *S. islandicus* strain 15a (si15a) collected in our lab was also used a control in the detection for smRNA sequence specificity hybridization. (D, E) Detection of miRNA candidate production in an *in vitro* reaction at 37°C (D) and 65°C (E) using *in vitro* precursor transcripts incubated with Ssp2 total extract. Synthetic 20-nt to 23-nt RNA oligos were used as size markers.

To search for potential *S. solfataricus* miRNA loci, we performed a computational screen to identify long transcripts from smRNA loci capable of folding into miRNA precursor-like molecules with imperfectly matched inverted repeats. The smRNAs generated from precursor sequences with hairpin-shaped secondary structure and the precursor sequences centered around small RNA production regions were considered as miRNA candidates. Using these criteria, 29 small RNAs were identified as putative miRNAs (Fig. 4A and supplemental Table S1). All candidate miRNAs had putative precursor sequences of 51–184-nt long with a single stem-loop structure (Fig. 4B and supplementary Fig. S1A), resembling the properties of eukaryotic miRNAs [60]. The miRNA candidates with several nucleotide variations at either end were sorted into the same family. Hypothetical proteins were the main source of these candidate precursors, followed by intergenic regions and other protein-coding genes (Supplemental Table S1). One candidate precursor matched to two inversely orientated ORFs in ISC1043, and one matched to the border of the overlapping region of neighboring genes (Sso2445 and Sso2448). These two were removed from the candidate list. Among the eight candidate precursors that mapped to protein-coding sequences, three were derived from antisense orientation, resembling non-coding RNAs derived from overlapping *cis*-antisense transcripts [44]. Interestingly, a large number of 22 to 25-nt smRNAs matched to a single position were found in one of the three potential non-coding RNAs (Sso0016/antisense) (Fig. 4A). This might suggest that this *cis*-antisense non-coding RNA regulates gene expression via a process reminiscent of the eukaryotic miRNA-mediated pathway. Therefore, this stem-loop sequence (Sso0016/antisense) and its coded smRNA were, kept in the candidate list (Supplemental Table S1). Analysis of the 20 sequences, including those derived from intergenic regions and hypothetical proteins as well as the Sso0016/antisense, revealed that the miRNA candidates were 18–26-nt in length and displayed a preference for adenosine at their 5′ ends (Supplementary Fig. S1B), which differs from the preference for uridine at the 5′ end in most eukaryotic miRNAs [60]. Eight miRNA candidate families had pairing star strand (*) or close variants with a lower cloning frequency sequenced (Supplemental Fig. S1A and Table S1), which provided strong evidence that they were processed from hairpin-shaped sequences by an RNase III-like enzyme as in eukaryotic organisms [51], [61], [62], [63].

The expression levels of the most frequently cloned miRNA candidates (supplemental Table S1) were readily detected by RNA blotting (e.g. candidate no. 1 and 5) (Fig. 4C). To determine whether the putative hairpin-shaped transcripts can be cleaved to generate miRNA candidates, we incubated an *in vitro* transcribed putative precursor RNA with total extract from Ssp2 culture and successfully detected the production of miRNA candidates in six of the seven tested putative precursors at 37°C (Fig. 4D, 4E). We also noted that a higher incubation temperature (65°C) was more efficient for the *in vitro* production of miRNA candidates for all seven transcripts tested (Fig. 4E). Indeed, the production of one of miRNAs candidates (no. 20) was only detected under the higher temperature reaction conditions (Fig. 4D). This suggests that the higher reaction temperature favors the enzyme activities in Ssp2 extracts, which is unsurprising given the hyperthermophilicity of Ssp2. Taken together, our results reveal that our identified miRNA candidates are derived from hairpin-shaped precursors.

### Prediction of miRNA candidate target genes

Eukaryotic miRNAs are known to pair with target mRNAs to regulate their expression [5], [64]. We searched for Ssp2 miRNA canditate targets among the annotated protein-coding transcripts using criteria modified from plant miRNA target prediction algorithms [14], [65]. Applying a cutoff penalty score of 4.0, a total of 18 non-transposase putative target genes were obtained for 5 Ssp2 miRNA canditates (no. 3, 3*, 14*, 19 and 20) (Supplemental Table S2). All of the putative miRNA candidate binding sites were located in the coding regions of the target genes, as have been found for most plant miRNAs, which direct site-specific cleavage of their target RNAs [66], [67].

**Figure 5:**
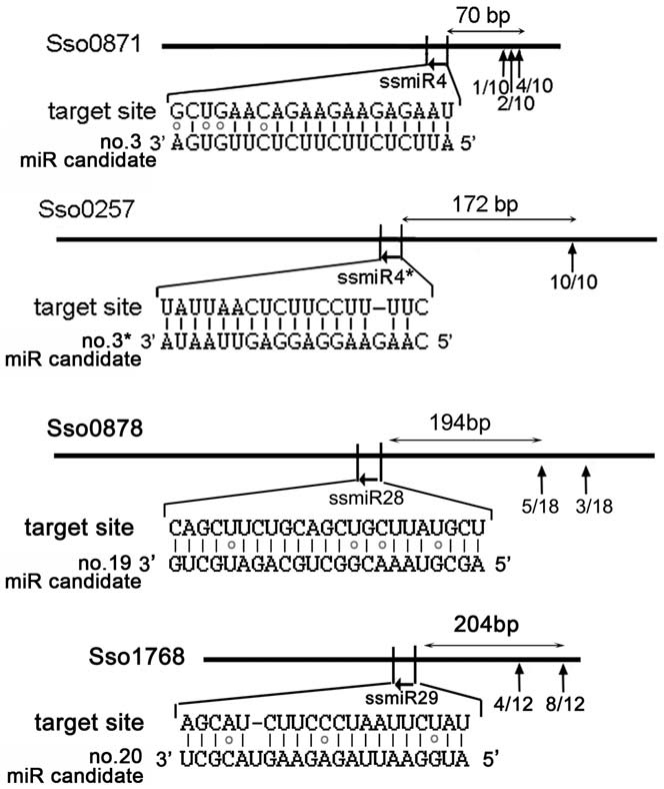
Alignment of *S. solfataricus* p2 miRNA candidates with their putative targets and mapping of the target cleavage sites. 5′ RACE PCR identified four predicted miRNA candidate targets (each ID is indicated). The regions and nucleotide sequences of the target sites that aligned with miRNA candidate (horizontal arrows) are shown. Vertical arrows (downstream of the miRNA candidate aligned regions) and numbers indicate the position and frequencies of 5′ termini of 3′ cleavage-truncated RNAs as determined by 5′ RACE. Two-way arrows show the distance from the target site to the most frequently mapped cleavage site.

To find out whether these miRNA candidates can induce the cleavage of target RNAs in Ssp2, we applied a modified 5′ rapid amplification of cDNA ends (RACE) assay to detect the predicted 3′cleavage products. We obtained a single PCR product from 4 of the 18 putative targets for 3 miRNA candidates (no. 3, 19 and 20) and one star strand (no. 4*). The cleavage sites, however, were all mapped to several to hundreds of basepairs downstream of the miRNA candidate aligned regions (Fig. 5). Interestingly, three of these four putative target RNAs (Sso0871, Sso0257 and Sso0878) were found to be included in an RNA group described in previous report [44]. This RNA group contains additional internal cleavage sites corresponding to positions where the RNAs are cleaved by endoribonucleases to promote RNA turnover/degradation [44]. To test whether the cleavage was mediated by miRNA candidates, we co-incubated the *in vitro* transcribed potential target RNA and the related miRNA candidate precursor RNA with Ssp2 total extract, and we detected the expression of mature miRNA candidate but failed to detect the expected miRNA candidates-mediated 3′ cleavage product (data not shown). The result might suggest a miRNA candidate unrelated unspecific degradation of mRNAs in Ssp2. However, we could not rule out that the total Ssp2 extract contains low levels of the active components required for miRNA candidate-mediated cleavage. Future studies should test cleavage activities using a different fractionated extract of the Ssp2 culture.

## Discussion

The diversity of small RNAs has been well demonstrated in multicellular and unicellular eukaryotic organisms but remains unknown in archaea. Here, we have shown that the hyperthermophilic archaeon *S. solfataricus* p2 (Ssp2) contains a variety of small RNAs, approximately 20-nt in length similar to those identified in eukaryotes. The cloned small RNAs include potential miRNAs, phased siRNAs and other siRNAs originated from intergenic regions, annotated protein-coding genes, repetitive sequences, regions producing noncoding RNAs (rRNAs and tRNAs), and snoRNAs, as well as smRNAs originating from CRISPR loci. The presence of these small RNAs indicates an unexpected complexity of eukaryotic RNAi-like processes in archaea.

We confirmed the expression of some of the predicted miRNA candidates in Ssp2 by RNA blotting (Fig. 4C), and the production of some miRNA candidates from *in vitro* transcribed putative precursor RNAs incubated with Ssp2 extract (Fig. 4D). Moreover, we noted that a higher incubation temperature was more suitable for the *in vitro* production of miRNAs from predicted precursor transcripts (Fig. 4E), a result consistent with the hyperthermophilicity of Ssp2. One of miRNA candidates (no. 1) derived from antisense of Sso0016, which encodes the transcription regulator (exsB) related protein, was coincident with the recent discovery of a large amount of *cis*-antisense non-coding RNAs and that suggested that antisense-based mechanisms might be a common regulatory process in Archaea [44]. The fact that this miRNA candidate family smRNAs were cloned from a single position in the precursor Sso0016/antisence transcript (Fig. 4A), together with the detection of miRNA candidate (no. 1) accumulation in Ssp2 (Fig. 4C) and in an *in vitro* assay using *in vitro* transcribed Sso0016/antisence precursor incubated with Ssp2 culture extract (Fig. 4D), suggest that the *cis*-antisense transcript of Sso0016 regulates the Sso0016 gene probably through the production and effect of the smRNA. Further analysis, such as the over-expression of the Sso0016/antisence transgene in Ssp2, will be helpful to address this issue. Nevertheless, we can conclude that active components of an RNase III-like enzyme for synthesis of smRNAs approximately 20-nt in length exist in Ssp2. ATP-dependent RNA helicase related proteins in Ssp2 contain both DEADc and HELICc superfamily multi-domains (Supplementary Fig. S2) and are highly similar to eukaryotic Dicer or DCL proteins [68]. Although Dicer activity remains to be elucidated, the Ssp2 ATP-dependent RNA helicase might be a component of the machinery responsible for Ssp2 smRNA synthesis.

The activities of miRNA candidate-mediated target cleavage might be low in the Ssp2 total extract. The fact that no miRNA candidates cofractionated with the site-specific cleavage products of their predicted targets in the 5′RACE assay (Fig. 5), suggest that the prevalent miRNA/target site-specific cleavage pathway found in most plant miRNAs may not exist in Ssp2. All of the related cleavage sites of the miRNA candidates mapped to downstream of the miRNA candidates/target aligned regions, suggesting that these miRNA candidates in Ssp2 may function similarly to some plant miRNAs (e.g., miR173, miR390 and miR828) in directing *TASs* transcript cleavage at regions out of the miRNA/target complementary site [14], [15], [16], [17]. Whether the mapped cleavage sites of the tested RNAs in Ssp2 (Fig. 5) depend on the related miRNA candidates as well as the biological significance of this cleavage need to be further investigated.

AGO-specialized and AGO-specific miRNAs are crucial factors required for miRNA-mediated cleavage in plants [69]. Some of the prokaryotic PAZ-domain containing AGO homologs (pAgos) are also shown to possess nuclease activity, and hypothesized to be key components of a novel class of prokaryotic immune system [70]. This implies a functional analogy with the prokaryotic CRISPR/Cas system and a direct evolutionary connection with eukaryotic RNAi [70]. We have systematically screened 71 fully sequenced archaeal genomes for the presence of AGO family proteins [50]. Five archaeal strains were found to encode Piwi-domains based on comparison with the previously reported *P. furiosus* AGO protein and twenty contained an RNase HI domain, which has a tertiary structure similar to that of the eukaryotic Piwi domain, but no PAZ domain was identified except in *P. furiosus*. However, there is no AGO/Piwi/PAZ domain protein in Ssp2. An RNase HI in *S. tokodaii* 7, another genus of *Sulfolobus*, was reported to cleave dsRNA [71], and a gene encoding a homolog of RNase HII was found in Ssp2. Whether the RNase HII and the DEADc domain-containing ATP-dependent RNA helicase proteins (Supplementary Fig. S2), other proteins, such as CRISPR crRNA-related Cas proteins in Ssp2 [35], [36], [37], or some Ssp2 proteins that exhibited *in vitro* endoribonucleolytic activity [44] are involved in the smRNA-mediated cleavage process resembling the plant miRNA/tasiRNA-like pathway remain to be determined. The observation that the ribosome component L7Ae-associated conserved noncoding RNA exhibited complementarity to the 3′UTR of a transposase mRNA encoded by Sso2103 [46], [48] led to the speculation that Archaea might employ an RNA-guided mechanism to silence gene expression and that L7Ae might be an integral component of archaeal RISC-like particles [72]. In addition to the host RNAi-like CRISPR-derived crRNA defense system [29], the restricted presence of the AGO and Dicer domains in some archaeal species [50] indicates that these or functionally related proteins may have emerged in some archaeal species and expanded during evolution in eukaryotes. In agreement with this, our results suggest that an endogenous gene regulation system analogous to the RNAi system in eukaryotes may exist in prokaryotes. Our findings provide evidence for smRNA (∼20-nt in length) and potential miRNAs in archaea and suggest that the siRNA/miRNA-related regulatory pathway may represent an ancient mechanism of gene regulation that evolved prior to the emergence of eukaryotic cells.

## Materials and Methods

### *Sulfolobus* strains and culture condition

*Solfolobus solfataricus* P2 and *Solfolobus islandicus* were grown aerobically at 80°C with shaking in Zillig's medium [73] supplemented with 0.2% sucrose and 0.05% yeast extract and adjusted to an initial pH of 3.1.

### DNA isolation

S. solfataricus P2 cells were harvested by centrifugation at 14,000 rpm for 15 min at 4°C, resuspended in 0.2–0.3 mL of 10 mM Tris-HCL, pH 8.0/1 mM EDTA/1%SDS/Protease K, and incubated at 50°C for 2 hours. This solution was extracted once each with equal volume of phenol, phenol/chloroform/isopentanol (25∶24∶1), and chloroform/isopentanol (24∶1). Sodium acetate (3 M, pH 5.2) was added to final aqueous phase to a concentration of 0.3 M, followed by DNA precipitation with 3 volumes of ice-cold ethanol at −20°C for 2 hours. DNA pellet was washed in 70% ethanol, air dried. and was dissolved in TE buffer [74].

### *S. solfataricus* extract preparation

*S. solfataricus* P2 cultures were collected at OD_600_ = 1.5 and homogenized by sonification in 20 mL extraction buffer (25 mM Tris-HCl, 25 mM KCl, 5 mM MgCl_2_ at pH 7.5) containing 2 mM DTT and 1 tablet/10 mL (Roche) protease inhibitor cocktail. Cell debris was removed by centrifugation at 12,000 rpm for 20 min at 4°C. The supernatant was collected.

### Small RNA preparation and cloning

Total RNA was extracted from *S. solfataricus* P2 cultures at OD_600_ = 1.0 in Zillig medium using Trizol (Invitrogen), and small RNAs were enriched by LiCl precipitation method. The isolated small RNAs were separated by 15% denaturing PAGE, and small RNAs of 17–30 nt were gel-purified. Small RNAs were ligated to a 5′adaptor and a 3′ acceptor sequentially, and thenamplified by RT–PCR as described [53]. PCR products were reamplified using a pair of Solexa cloning primers, for sequencing by BGI, Shenzhen as previously described [75].

### Small RNA analysis

After removing adaptor/acceptor sequences from the raw reads obtained using Solexa sequencing technology, the remaining small RNA sequences were mapped to the *S. solfataricus* P2 genome using Perl scripts. Relationships of small RNAs to annotated genes were determined by comparing the genomic loci of small RNAs with those of genes. Small RNAs derived from known noncoding RNAs were identified by comparing small RNAs with the sequences of noncoding RNAs collected in Rfam (http://www.sanger.ac.uk/Software/Rfam) [76].

### Predictions of poteintial miRNAs

The prediction of *S. solfataricus* P2 poteintial miRNAs was carried out using Perl script with criteria similar to those applied for Arabidopsis miRNA prediction [77] and subjected to RNA secondary structure check using Mfold [78].

### Prediction of miRNA candidate targets

We modified criteria that were developed for plant miRNA target prediction to predict targets of *S. solfataricus* P2 miRNA candidates [14], [65]. The putative target sites of all miRNA candidates were identified by aligning miRNA candidate sequences to the annotated gene sequences of *S. solfataricus* P2 using Perl script.

### Validation of small RNA

Total RNA was extracted from *S. solfataricus* P2 and *S. islandicus* at OD_600_ = 0.3 using Trizol extraction (Invitrogen). High molecular weight RNA was selectively precipitated from the total RNA by addition of one volume of 4 M LiCl. The low molecular weight RNA was precipitated with three volumes of ethanol and dissolved in nuclease-free water. The resulting low-molecular-weight-enriched RNA was separated by electrophoresis on denaturing 17% polyacrylamide gels and electrically transferred to Hybond-N+ membranes. Blots were hybridized with oligonucleotide probes that were end-labeled with γ-^32^P-ATP using T4 kinase (NEB). Hybridization signal intensity was measured using a Phosphor-Imager (GE Healthcare).

Probes used for miRNA candidates are listed below:

no. 1p: 5′ GGTGCCCCACTACATTTGACCT 3′

no. 4p: 5′ TAAGTAGCCCCTACATGAACT 3′

no. 5p: 5′ CGTGGCTCCACCTAAACCGGTT 3′

no. 9p: 5′ TGAGGGTATGTCTTATTCATCCT 3′

no. 15p: 5′ AGACAGATCTCATCACCTTC 3′

no. 17p: 5′ CTTTTTATTCATTCGATTCCCTC 3′

no. 19p: 5′ CAGCATCTGCAGCCGTTTACGCT 3′

no. 20p: 5′ AGCGTACTTCTCTAATTCCAT 3′

### Validation of miRNA candidate target

The FirstChoice RLM-RACE kit (Ambion) was used for RLMRACE assays according to the manufacturer's instructions. For analysis of miRNA candidate-guided cleavage sites, the RNA Oligo adaptor was directly ligated to total RNA without calf intestinal phosphatase treatment. For the first round of nested PCR, the 5′RACE Outer Primer together with gene-specific outer primerswere used:

5′ RACE outer primer 5′ GCTGATGGCGATGAATGAACACTG 3′

Sso0871-outer 5′ TATTTTCAGCTTGATCCCAGACC 3′

Sso0257-outer 5′ ACTCCAGAGATCAGCAAACCTAC 3′

Sso0878-outer 5′ CGGTAACTATTGGTGATGCG 3′

Sso1768-outer 5′ CCAACCAGCATTAATCTTGTTG 3′

For the second round of nested PCR, the 5′RACE Inner Primer and gene-specific inner primers are listed below.

5′ RACE inner primer 5′ CGCGGATCCGAACACTGCGTTTGCTGGCTTTGATG 3′

Sso0871-inner 5′ GATCCCAGACCGCACTTTTAGA 3′

Sso0257-inner 5′ TCGCTTTCTATTAAAGATCTAACAA 3′

Sso0878-inner 5′ CTTGGATTCCAATCATTCTAGG 3′

Sso1768-inner 5′ TCCTCCTACAGACGCTAATGAC 3′

Gel-purified PCR products were then cloned into pGM-T vector (TIANGEN) for sequencing.

### Plasmids construction

Predicted premiRNAs sequences were amplified from *S. solfataricus* P2 genome by PCR using eight pairs of primers, respectively:

no. 1pfor 5′ CTGGAGTCCCACAATGTTTTTGCCCACCTT 3′

no. 1prev 5′ GGATCCTGGTCCAAACAAGAGAATTGAGATA 3′

no. 4pfor 5′ CTCGAGTTAGGTTAGTTCATGTAGGG 3′

no. 4prev 5′ GGATCCCCTAATTCATAAAGGCTACC 3′

no. 9pfor 5′ GGAATATCAAGCAAAAAAGAGAA 3′

no. 9prev 5′ TTACCTCAACATTATCGATCTAAAG 3′

no. 15pfor 5′ CTTCAAATATGACGAAAACATAGAG 3′

no. 15prev 5′ GAATGGCTGAAATCTTCCTG 3′

no. 17pfor 5′ CAATAAGAAAAATGTTGATGAAGAA 3′

no. 17prev 5′ TTAACCATAATTGATAACTGGACAA 3′

no. 19pfor 5′ AAAAGTTGGAATATTATGCAATAGT 3′

no. 19prev 5′ TGCTATCATAACGTTATATACTCCTC 3′

no. 20pfor 5′ TTCTCTTATTATTAGAATTGTACGC 3′

no. 20prev 5′ CACAAAGTTAGCATAATCCTCAA 3′

The PCR products were then cloned into pGM-T Vector (TIANGEN), producing pT-no. 1p, pT-no. 4p, pT- no. 9p, pT-no. 15p, pT- no. 17p, pT- no. 19p and pT-no. 20p, which were verified by sequencing.

### *In Vitro* Transcription and predicted premiRNA splicing

To prepare in vitro transcripts of the predicted premiRNAs, plasmids pT-no. 1p, pT-no. 4p, pT- no. 9p, pT-no. 15p, pT- no. 17p, pT- no. 19p and pT-no. 20p, were linearized by NcoI/SpeI (Takara) and purified by Wizard DNA Clean-up system(Promega) and used as templates for *in vitro* transcription using the T7/SP6 in vitro transcription system(Promega) following the manufacturer's directions. After in vitro transcription, the DNA templates were removed by digestion with RNase-free DNase I. Transcripts of predicted premiRNAs were incubated with Ssp2 total extracts at 37°C and 65°C in 20 mM HEPES (pH 7.0), 250 mM KCl, 1.5 mM MgCl_2_,1 mM ATP, 10 mM DTT, in the presence of 1 unit of ribonuclease inhibitor (Takara ) for 20 min [25], and then denatured at 99°C for 5 min. The resulting RNAs were separated by electrophoresis on denaturing 10% polyacrylamide gels and electrically transferred to Hybond-N+ membranes.

## Supporting Information

Figure S1 *S. solfataricus* p2 miRNAs candidates and their putative precursor structures. (A) Secondary structures of miRNA candidate precursors. The IDs of the precursor loci and their lengths are indicated. The sequences corresponding to the mature miRNA candidates (the most frequently cloned sequence in an miRNA candidate family) are shown in red and the star strand (*) candidate in pink. The precursor sequences were folded using the mfold (v3.2) program. (B) The size distribution of the Ssp2 miRNA candidates and the sequence composition of their 5′ end are shown. The redundant and unique smRNA reads are represented as white and black bars, respectively.
(JPG)

Figure S2 Conserved domains in ATP-dependent RNA helicases in *S. solfataricus* p2. The protein IDs and conserved DEAD-box and HELIC-family domains residues in the helicase proteins are indicated. The representative sequence alignments with a significantly low E-Value between these helicases and Arabidopsis DCL1–4 are also shown.
(TIF)

Table S1 Candidate miRNAs and their putative precursor loci in *S. solfataricus*.
(DOC)

Table S2 Predicted miRNA candidates targets.
(DOC)
