# Supplementary figures and images for: Identification and Characterization of Small RNAs in the Hyperthermophilic Archaeon Sulfolobus solfataricus
Source: PLoS One. 2012 Apr 13;7(4):e35306. doi: 10.1371/journal.pone.0035306 (PMC3325985; doi:10.1371/journal.pone.0035306)

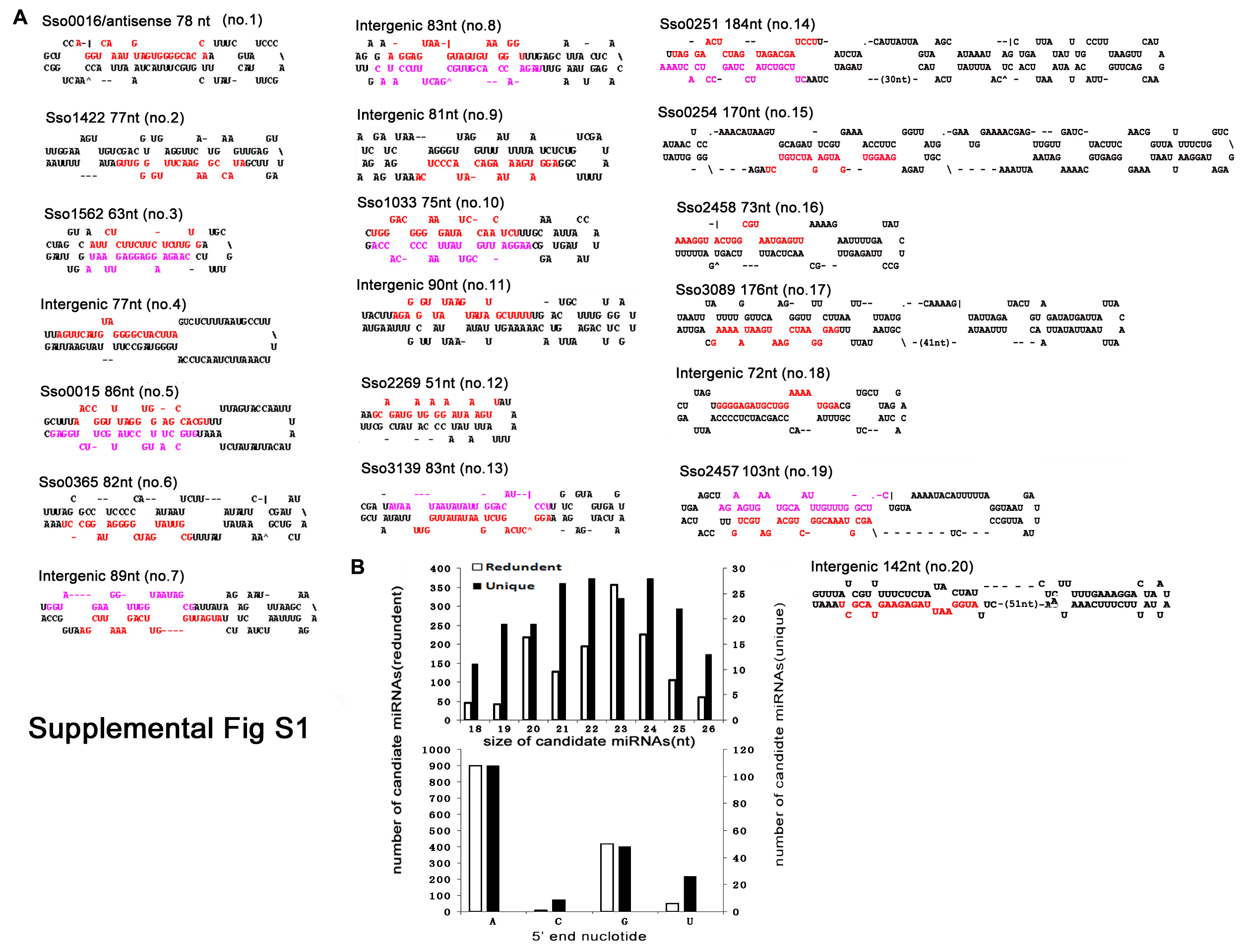

Supplement: Figure S1 — S. solfataricus p2 miRNAs candidates and their putative precursor structures. (A) Secondary structures of miRNA candidate precursors. The IDs of the precursor loci and their lengths are indicated. The sequences corresponding to the mature miRNA candidates (the most frequently cloned sequence in an miRNA candidate family) are shown in red and the star strand (*) candidate in pink. The precursor sequences were folded using the mfold (v3.2) program. (B) The size distribution of the Ssp2 miRNA candidates and the sequence composition of their 5′ end are shown. The redundant and unique smRNA reads are represented as white and black bars, respectively. (JPG) [file pone.0035306.s001.jpg]

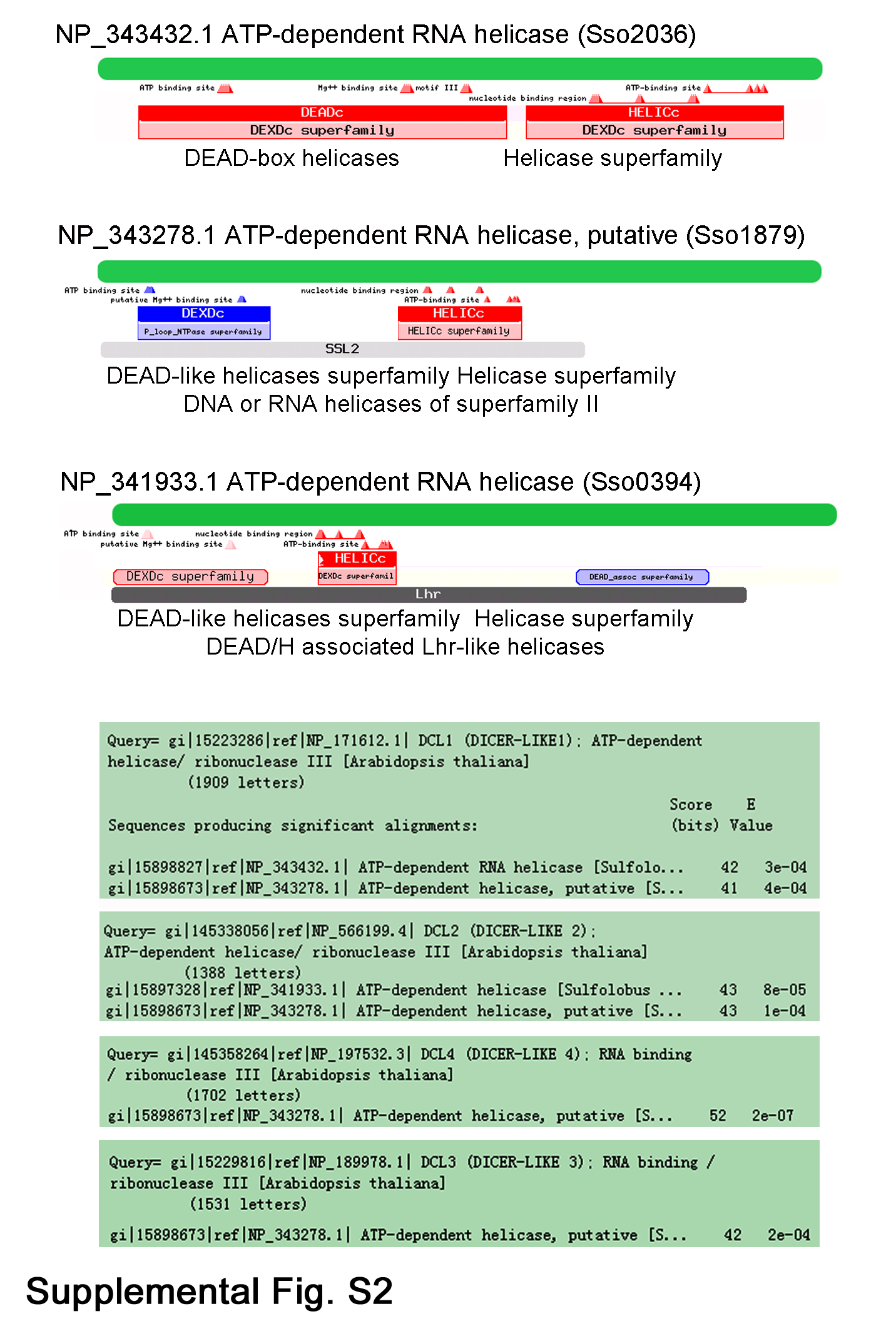

Supplement: Figure S2 — Conserved domains in ATP-dependent RNA helicases in S. solfataricus p2. The protein IDs and conserved DEAD-box and HELIC-family domains residues in the helicase proteins are indicated. The representative sequence alignments with a significantly low E-Value between these helicases and Arabidopsis DCL1–4 are also shown. (TIF) [file pone.0035306.s002.tif]
