# Supplementary material for: Identification and Characterization of Small RNAs in the Hyperthermophilic Archaeon Sulfolobus solfataricus
Source: PLoS One. 2012 Apr 13;7(4):e35306. doi: 10.1371/journal.pone.0035306 (PMC3325985; doi:10.1371/journal.pone.0035306)
Supplement: Table S1 — Candidate miRNAs and their putative precursor loci in S. solfataricus. (DOC) [file pone.0035306.s003.doc]

Supplemental Table S1. Candidate miRNAs and their putative precursor loci in *S. solfataricus*

| Precursor | Origin | Start | End | Strand | Pos^a^ | miRNA | Sequence(5’-3’) | Len^b^ | Reads |
| --- | --- | --- | --- | --- | --- | --- | --- | --- | --- |
| 1 | Sso0016  13920-15314 (-) Transcription regulator (exsB) related protein | 14031 | 14109 | + | 5’ | t0149811 | CAGGTCAAATGTAGTGGGGCACCA | 24 | 3 |
|  |  |  |  |  |  | t0264563 | AGGTCAAATGTAGTGGGGCAC | 21 | 2 |
|  |  |  |  |  |  | t0009589 | AGGTCAAATGTAGTGGGGCACC | 22 | 54 |
|  |  |  |  |  |  | t0008553 | AGGTCAAATGTAGTGGGGCACCA | 23 | 60 |
|  |  |  |  |  |  | t0018834 | AGGTCAAATGTAGTGGGGCACCAA | 24 | 27 |
|  |  |  |  |  |  | t0024398 | AGGTCAAATGTAGTGGGGCACCAAT | 25 | 21 |
|  |  |  |  |  |  | t0210574 | AGGTCAAATGTAGTGGGGCACCAATT | 26 | 2 |
|  |  |  |  |  |  | t0374905 | GGTCAAATGTAGTGGGGCACCA | 22 | 1 |
| 2 | Sso1422  Hypothetical protein | 1276843 | 1276919 | + | 3’ | t0157367 | ATACCGAAGAACTTTGGGGTTG | 22 | 3 |
| 3 | Sso1562  Hypothetical protein | 1408023 | 1408085 | + | 5’ | t1027935 | ATTCTCTTCTTCTCTTGTG | 19 | 1 |
|  |  |  |  |  |  | t0205743 | ATTCTCTTCTTCTCTTGTGA | 20 | 2 |
|  |  |  |  |  |  | t0542945 | ATTCTCTTCTTCTCTTGTGAT | 21 | 1 |
|  |  |  |  |  | 3’ | t0422506 | CAAGAAGGAGGAGTTAATA | 19 | 1 |
|  |  |  |  |  | 3’ | t0137866 | AGGTTTCTGGGATGCCTCCT | 20 | 3 |
| 4 | Intergenic | 2054479 | 2054555 | + | 5’ | t0149096 | TTAGTTCATGTAGGGGCT | 18 | 3 |
|  |  |  |  |  |  | t0158663 | TTAGTTCATGTAGGGGCTA | 19 | 3 |
|  |  |  |  |  |  | t0436628 | TAGTTCATGTAGGGGCTAC | 19 | 1 |
|  |  |  |  |  |  | t1280180 | TAGTTCATGTAGGGGCTACT | 20 | 1 |
|  |  |  |  |  |  | t0362082 | AGTTCATGTAGGGGCTACT | 19 | 1 |
|  |  |  |  |  |  | t0217115 | AGTTCATGTAGGGGCTACTT | 20 | 2 |
|  |  |  |  |  |  | t0124448 | AGTTCATGTAGGGGCTACTTA | 21 | 4 |
|  |  |  |  |  |  | t1166747 | AGTTCATGTAGGGGCTACTTAG | 22 | 1 |
|  |  |  |  |  |  | t0125171 | AGTTCATGTAGGGGCTACTTAGTC | 24 | 4 |
|  |  |  |  |  |  | t0282177 | AGTTCATGTAGGGGCTACTTAGTCT | 25 | 2 |
|  |  |  |  |  |  | t1414381 | CATGTAGGGGCTACTTAGT | 19 | 1 |
|  |  |  |  |  | 3’ | t0836663 | TTAATGCCTTTTCAAATT | 18 | 1 |
| 5 | Sso0015  Hypothetical protein | 13729 | 13814 | - | 5’ | t1457950 | AACCGGTTTAGGTGGAGCC | 19 | 1 |
|  |  |  |  |  |  | t0353019 | AACCGGTTTAGGTGGAGCCA | 20 | 1 |
|  |  |  |  |  |  | t0655111 | AACCGGTTTAGGTGGAGCCAC | 21 | 1 |
|  |  |  |  |  |  | t0017459 | AACCGGTTTAGGTGGAGCCACG | 22 | 30 |
|  |  |  |  |  |  | t0003073 | AACCGGTTTAGGTGGAGCCACGT | 23 | 152 |
|  |  |  |  |  |  | t0006551 | AACCGGTTTAGGTGGAGCCACGTT | 24 | 77 |
|  |  |  |  |  |  | t0093637 | AACCGGTTTAGGTGGAGCCACGTTT | 25 | 5 |
|  |  |  |  |  |  | t0154889 | AACCGGTTTAGGTGGAGCCACGTTTT | 26 | 3 |
|  |  |  |  |  |  | t0155122 | ACCGGTTTAGGTGGAGCCACGT | 22 | 3 |
|  |  |  |  |  |  | t1143019 | ACCGGTTTAGGTGGAGCCACGTT | 23 | 1 |
|  |  |  |  |  |  | t0937519 | CCGGTTTAGGTGGAGCCACGT | 21 | 1 |
|  |  |  |  |  | 3’ | t1478014 | ATGTGCCTATTGCCTATGC | 19 | 1 |
|  |  |  |  |  |  | t1416748 | GTGCCTATTGCCTATGCTTCTGGAG | 25 | 1 |
|  |  |  |  |  |  | t0769793 | ATTGCCTATGCTTCTGGAG | 19 | 1 |
| 6 | Sso0365  Hypothetical protein | 310515 | 310596 | - | 3’ | t0803622 | GCGTTATGATCGGGGATAGGCC | 22 | 1 |
|  |  |  |  |  |  | t0042154 | GCGTTATGATCGGGGATAGGCCT | 23 | 11 |
|  |  |  |  |  |  | t0041690 | GCGTTATGATCGGGGATAGGCCTA | 24 | 11 |
|  |  |  |  |  |  | t1608389 | GCGTTATGATCGGGGATAGGCCTAA | 25 | 1 |
|  |  |  |  |  |  | t1165298 | CGTTATGATCGGGGATAGGCCT | 22 | 1 |
|  |  |  |  |  |  | t1388259 | GTTATGATCGGGGATAGGCCTA | 22 | 1 |
|  |  |  |  |  |  | t0786661 | GTTATGATCGGGGATAGGCCTAAA | 24 | 1 |
|  |  |  |  |  |  | t1535889 | TTATGATCGGGGATAGGCCTAAAT | 24 | 1 |
|  |  |  |  |  |  | t0397611 | ATGATCGGGGATAGGCCTAAATCT | 24 | 1 |
| 7 | Intergenic | 2973044 | 2973132 | - | 5’ | t1561664 | GGTAGAAGGTTGGTAATAGCG | 21 | 1 |
|  |  |  |  |  | 3’ | t1588362 | ATGATTGGTTCAGAAATT | 18 | 1 |
|  |  |  |  |  |  | t0933842 | ATGATTGGTTCAGAAATTCG | 20 | 1 |
|  |  |  |  |  |  | t0177288 | ATGATTGGTTCAGAAATTCGA | 21 | 3 |
|  |  |  |  |  |  | t0327290 | ATGATTGGTTCAGAAATTCGAATGGC | 26 | 2 |
|  |  |  |  |  |  | t0695737 | TGATTGGTTCAGAAATTCGAATGGC | 25 | 1 |
| 8 | Intergenic | 676840 | 676922 | - | 5’ | t1231162 | GAAGGAGTAAGTAGTGTAAGGGGT | 24 | 1 |
|  |  |  |  |  |  | t0930947 | GAAGGAGTAAGTAGTGTAAGGGGTT | 25 | 1 |
|  |  |  |  |  |  | t0068489 | AAGGAGTAAGTAGTGTAAGGGGT | 23 | 7 |
|  |  |  |  |  |  | t0218736 | AAGGAGTAAGTAGTGTAAGGGGTT | 24 | 2 |
|  |  |  |  |  |  | t0060428 | AGGAGTAAGTAGTGTAAGGGGT | 22 | 8 |
|  |  |  |  |  |  | t0095822 | AGGAGTAAGTAGTGTAAGGGGTT | 23 | 5 |
|  |  |  |  |  | 3’ | t1402862 | TTAGAACCACGTTGCGACTTTCCATA | 26 | 1 |
|  |  |  |  |  |  | t0089774 | TAGAACCACGTTGCGACTTTCCATAC | 26 | 5 |
|  |  |  |  |  |  | t1669084 | AGAACCACGTTGCGACTTTCC | 21 | 1 |
|  |  |  |  |  |  | t1268953 | AGAACCACGTTGCGACTTTCCA | 22 | 1 |
|  |  |  |  |  |  | t0778533 | AGAACCACGTTGCGACTTTCCATAC | 25 | 1 |
|  |  |  |  |  |  | t0400705 | AACCACGTTGCGACTTTCCAT | 21 | 1 |
|  |  |  |  |  |  | t0666394 | AACCACGTTGCGACTTTCCATAC | 23 | 1 |
| 9 | Intergenic | 815404 | 815484 | - | 3’ | t1371937 | CGGAGGATGAATAAGACAT | 19 | 1 |
|  |  |  |  |  |  | t1170864 | GGAGGATGAATAAGACATACCCTCAA | 26 | 1 |
|  |  |  |  |  |  | t0217158 | GGATGAATAAGACATACCCTCAAA | 24 | 2 |
|  |  |  |  |  |  | t0563558 | GAGGATGAATAAGACATAC | 19 | 1 |
|  |  |  |  |  |  | t1389114 | AGGATGAATAAGACATACC | 19 | 1 |
|  |  |  |  |  |  | t0162534 | AGGATGAATAAGACATACCCT | 21 | 3 |
|  |  |  |  |  |  | t0203389 | AGGATGAATAAGACATACCCTC | 22 | 2 |
|  |  |  |  |  |  | t0034207 | AGGATGAATAAGACATACCCTCA | 23 | 14 |
|  |  |  |  |  |  | t0053239 | AGGATGAATAAGACATACCCTCAA | 24 | 9 |
|  |  |  |  |  |  | t0040056 | AGGATGAATAAGACATACCCTCAAA | 25 | 12 |
|  |  |  |  |  |  | t0060778 | AGGATGAATAAGACATACCCTCAAAT | 26 | 8 |
|  |  |  |  |  |  | t0228667 | GATGAATAAGACATACCCTCAAA | 23 | 2 |
|  |  |  |  |  |  | t0470747 | ATGAATAAGACATACCCTCAAA | 22 | 1 |
|  |  |  |  |  |  | t1088185 | ATGAATAAGACATACCCTCAAATG | 24 | 1 |
| 10 | Sso1033  Hypothetical protein | 893227 | 893301 | - | 5’ | t1541956 | TGGGACGGGAAGATATCCAAC | 21 | 1 |
|  |  |  |  |  |  | t0558355 | TGGGACGGGAAGATATCCAACT | 22 | 1 |
|  |  |  |  |  |  | t0079969 | TGGGACGGGAAGATATCCAACTCT | 24 | 6 |
|  |  |  |  |  |  | t1193437 | TGGGACGGGAAGATATCCAACTCTT | 25 | 1 |
|  |  |  |  |  | 3’ | t0214039 | AAGGATTGCGTTATTAACCCCACCA | 25 | 2 |
|  |  |  |  |  |  | t0203890 | AAGGATTGCGTTATTAACCCCACCAG | 26 | 2 |
|  |  |  |  |  |  | t0491831 | AGGATTGCGTTATTAACCCCACCAGA | 26 | 1 |
|  |  |  |  |  |  | t0646058 | TGCGTTATTAACCCCACCAGA | 21 | 1 |
| 11 | Intergenic | 1051864 | 1051953 | - | 5’ | t1611156 | AGAGGGTTATAAGTATATGCT | 21 | 1 |
|  |  |  |  |  |  | t0188288 | AGAGGGTTATAAGTATATGCTTTT | 24 | 2 |
|  |  |  |  |  |  | t0351684 | GAGGGTTATAAGTATATGCT | 20 | 1 |
|  |  |  |  |  |  | t1282913 | GAGGGTTATAAGTATATGCTTTTTG | 25 | 1 |
|  |  |  |  |  | 3’ | t1647017 | GTGGATTGCTTCAGAATTG | 19 | 1 |
|  |  |  |  |  |  | t0548204 | GTGGATTGCTTCAGAATTGT | 20 | 1 |
|  |  |  |  |  |  | t0455723 | GTGGATTGCTTCAGAATTGTACAAAA | 26 | 1 |
|  |  |  |  |  |  | t1346451 | GGATTGCTTCAGAATTGTACAAAAA | 25 | 1 |
|  |  |  |  |  |  | t0613119 | CTTCAGAATTGTACAAAAAG | 20 | 1 |
| 12 | Sso2269  Hypothetical protein | 2080634 | 2080684 | - | 5’ | t1668655 | AGCAGATGATGAGGAATA | 18 | 1 |
|  |  |  |  |  |  | t0193889 | AGCAGATGATGAGGAATAAAGTT | 23 | 2 |
|  |  |  |  |  |  | t0939302 | AGCAGATGATGAGGAATAAAGTTA | 24 | 1 |
|  |  |  |  |  |  | t0130295 | AGCAGATGATGAGGAATAAAGTTATA | 26 | 3 |
|  |  |  |  |  |  | t0594520 | GCAGATGATGAGGAATAAA | 19 | 1 |
|  |  |  |  |  |  | t0212590 | GCAGATGATGAGGAATAAAGT | 21 | 2 |
|  |  |  |  |  |  | t0143684 | GCAGATGATGAGGAATAAAGTT | 22 | 3 |
|  |  |  |  |  |  | t0895867 | GCAGATGATGAGGAATAAAGTTA | 23 | 1 |
|  |  |  |  |  |  | t0728183 | GCAGATGATGAGGAATAAAGTTATA | 25 | 1 |
| 13 | Sso3139  Hypothetical protein | 2887330 | 2887412 | - | 5’ | t1481511 | ATAATAATATATTGGACATCCT | 22 | 1 |
|  |  |  |  |  | 3’ | t0147765 | AGAAAGGCTCAGTCTGAATAT | 21 | 3 |
|  |  |  |  |  |  | t1258426 | AGAAAGGCTCAGTCTGAATATA | 22 | 1 |
|  |  |  |  |  |  | t1105677 | AGAAAGGCTCAGTCTGAATATAT | 23 | 1 |
|  |  |  |  |  |  | t0123919 | AAGGCTCAGTCTGAATATATTGGTT | 25 | 4 |
|  |  |  |  |  |  | t0058520 | AAGGCTCAGTCTGAATATATTGGTTT | 26 | 8 |
|  |  |  |  |  |  | t0093577 | AGGCTCAGTCTGAATATATTGGT | 23 | 5 |
|  |  |  |  |  |  | t0011184 | AGGCTCAGTCTGAATATATTGGTT | 24 | 47 |
|  |  |  |  |  |  | t0016298 | AGGCTCAGTCTGAATATATTGGTTT | 25 | 32 |
|  |  |  |  |  |  | t0022150 | AGGCTCAGTCTGAATATATTGGTTTT | 26 | 23 |
| 14 | Sso0251  hypothetical protein | 217498 | 217681 | + | 5’ | t0817280 | TAGGAACTCTAGTAGACGAT | 20 | 1 |
|  |  |  |  |  |  | t0904082 | TAGGAACTCTAGTAGACGATCC | 22 | 1 |
|  |  |  |  |  |  | t0041671 | TAGGAACTCTAGTAGACGATCCT | 23 | 11 |
|  |  |  |  |  |  | t0258650 | TAGGAACTCTAGTAGACGATCCTT | 24 | 2 |
|  |  |  |  |  |  | t1425968 | AGGAACTCTAGTAGACGATCCTT | 23 | 1 |
|  |  |  |  |  | 3’ | t1050819 | CTTCGTCTATCCTAGCCTCACTAAA | 18 | 1 |
| 15 | Sso0254  hypothetical protein | 219879 | 220048 | + | 5’ | t0956630 | ACCTTCGGTTATGGAATGGAA | 21 | 1 |
|  |  |  |  |  |  | t0649207 | TCGGTTATGGAATGGAAAACGAGT | 24 | 1 |
|  |  |  |  |  |  | t0793003 | TGGAATGGAAAACGAGTT | 18 | 1 |
|  |  |  |  |  | 3’ | t0744151 | AGAGAAGGTGATGAGATCTGTCT | 23 | 1 |
|  |  |  |  |  |  | t0361402 | AGAGAAGGTGATGAGATCTGTCTAG | 25 | 1 |
|  |  |  |  |  |  | t1469255 | AGAAGGTGATGAGATCTGT | 19 | 1 |
|  |  |  |  |  |  | t0130189 | AGAAGGTGATGAGATCTGTC | 20 | 3 |
|  |  |  |  |  |  | t0035582 | AGAAGGTGATGAGATCTGTCT | 21 | 14 |
|  |  |  |  |  |  | t0031333 | AGAAGGTGATGAGATCTGTCTA | 22 | 16 |
|  |  |  |  |  |  | t0037689 | AGAAGGTGATGAGATCTGTCTAG | 23 | 13 |
|  |  |  |  |  |  | t0355365 | AGAAGGTGATGAGATCTGTCTAGA | 24 | 1 |
|  |  |  |  |  |  | t0202278 | AGAAGGTGATGAGATCTGTCTAGAG | 25 | 2 |
|  |  |  |  |  |  | t0125379 | GAAGGTGATGAGATCTGT | 18 | 4 |
|  |  |  |  |  |  | t0076487 | GAAGGTGATGAGATCTGTC | 19 | 6 |
|  |  |  |  |  |  | t0002953 | GAAGGTGATGAGATCTGTCT | 20 | 158 |
|  |  |  |  |  |  | t0022523 | GAAGGTGATGAGATCTGTCTA | 21 | 23 |
|  |  |  |  |  |  | t0058091 | GAAGGTGATGAGATCTGTCTAG | 22 | 8 |
|  |  |  |  |  |  | t0177475 | GAAGGTGATGAGATCTGTCTAGA | 23 | 3 |
|  |  |  |  |  |  | t0048188 | GAAGGTGATGAGATCTGTCTAGAG | 24 | 10 |
|  |  |  |  |  |  | t0047588 | GAAGGTGATGAGATCTGTCTAGAGG | 25 | 10 |
|  |  |  |  |  |  | t0165743 | AGGTGATGAGATCTGTCT | 18 | 3 |
|  |  |  |  |  |  | t1177300 | AGGTGATGAGATCTGTCTAG | 20 | 1 |
|  |  |  |  |  |  | t1268732 | AGGTGATGAGATCTGTCTAGA | 21 | 1 |
|  |  |  |  |  |  | t0699093 | AGGTGATGAGATCTGTCTAGAG | 22 | 1 |
|  |  |  |  |  |  | t0292200 | AGGTGATGAGATCTGTCTAGAGG | 23 | 2 |
|  |  |  |  |  |  | t0276129 | AGGTGATGAGATCTGTCTAGAGGG | 24 | 2 |
|  |  |  |  |  |  | t1197447 | GTGATGAGATCTGTCTAGAGGG | 22 | 1 |
|  |  |  |  |  |  | t0302799 | GTGATGAGATCTGTCTAGAGGGT | 23 | 2 |
|  |  |  |  |  |  | t0794937 | GATGAGATCTGTCTAGAGGGT | 21 | 1 |
| 16 | Sso2458  Conserved hypothetical protein | 2227872 | 2227944 | + | 5’ | t0420933 | TCAAAGGTACTGGCGTAATGAGTT | 24 | 1 |
|  |  |  |  |  |  | t0265057 | AAAGGTACTGGCGTAATGAGTT | 22 | 2 |
|  |  |  |  |  |  | t0304445 | AAGGTACTGGCGTAATGAGT | 20 | 2 |
|  |  |  |  |  |  | t0985335 | AAGGTACTGGCGTAATGAGTT | 21 | 1 |
|  |  |  |  |  |  | t0827323 | AAGGTACTGGCGTAATGAGTTA | 22 | 1 |
|  |  |  |  |  |  | t1191291 | AAGGTACTGGCGTAATGAGTTAA | 23 | 1 |
| 17 | Sso3089  hypothetical protein | 2842845 | 2843020 | + | 3’ | t0365906 | TGAGGGAATCGAATGAATAAAAAG | 24 | 1 |
|  |  |  |  |  |  | t0077167 | GAGGGAATCGAATGAATAA | 19 | 6 |
|  |  |  |  |  |  | t0215208 | GAGGGAATCGAATGAATAAA | 20 | 2 |
|  |  |  |  |  |  | t0346685 | GAGGGAATCGAATGAATAAAA | 21 | 1 |
|  |  |  |  |  |  | t0895231 | GAGGGAATCGAATGAATAAAAA | 22 | 1 |
|  |  |  |  |  |  | t0073024 | GAGGGAATCGAATGAATAAAAAG | 23 | 6 |
|  |  |  |  |  |  | t0997780 | GAGGGAATCGAATGAATAAAAAGC | 24 | 1 |
|  |  |  |  |  |  | t0553707 | GAGGGAATCGAATGAATAAAAAGCA | 25 | 1 |
|  |  |  |  |  |  | t0986849 | AGGGAATCGAATGAATAA | 18 | 1 |
|  |  |  |  |  |  | t0716746 | AGGGAATCGAATGAATAAAAAGCA | 24 | 1 |
| 18 | Intergenic | 1548440 | 1548511 | - | 5’ | t1609373 | TGGGGAGATGCTGGAAAATG | 20 | 1 |
|  |  |  |  |  |  | t1089339 | TGGGGAGATGCTGGAAAATGG | 21 | 1 |
|  |  |  |  |  |  | t0018074 | GGGGAGATGCTGGAAAAT | 18 | 29 |
|  |  |  |  |  |  | t0041565 | GGGGAGATGCTGGAAAATG | 19 | 12 |
|  |  |  |  |  |  | t0014874 | GGGGAGATGCTGGAAAATGG | 20 | 35 |
|  |  |  |  |  |  | t0011261 | GGGGAGATGCTGGAAAATGGA | 21 | 46 |
|  |  |  |  |  | 3’ | t1257651 | CCCAGCATCTCCCCAATT | 18 | 1 |
| 19 | Sso2457  hypothetical protein | 2226702 | 2226804 | - | 5’ | t1604089 | AGAAGTGAATGCAATTTGTTT | 21 | 1 |
|  |  |  |  |  |  | t0986764 | AGAAGTGAATGCAATTTGTTTGGCT | 25 | 1 |
|  |  |  |  |  |  | t1275568 | TTGTTTGGCTCTGTAAAAAA | 20 | 1 |
|  |  |  |  |  | 3’ | t1434286 | AGCGTAAACGGCTGCAGAT | 19 | 1 |
|  |  |  |  |  |  | t0333023 | AGCGTAAACGGCTGCAGATG | 20 | 2 |
|  |  |  |  |  |  | t0078097 | AGCGTAAACGGCTGCAGATGC | 21 | 6 |
|  |  |  |  |  |  | t0010841 | AGCGTAAACGGCTGCAGATGCT | 22 | 48 |
|  |  |  |  |  |  | t0009876 | AGCGTAAACGGCTGCAGATGCTG | 23 | 52 |
|  |  |  |  |  |  | t0055344 | AGCGTAAACGGCTGCAGATGCTGT | 24 | 8 |
| 20 | Intergenic | 1541561 | 1541702 | + | 3’ | t0128120 | ATGGAATTAGAGAAGTACGC | 20 | 3 |
|  |  |  |  |  |  | t0083352 | ATGGAATTAGAGAAGTACGCT | 21 | 5 |
|  |  |  |  |  |  | t0260499 | ATGGAATTAGAGAAGTACGCTA | 22 | 2 |
|  |  |  |  |  |  | t0302065 | ATGGAATTAGAGAAGTACGCTAA | 23 | 2 |
|  |  |  |  |  |  | t0315294 | ATGGAATTAGAGAAGTACGCTAAA | 24 | 2 |
|  |  |  |  |  |  | t0181583 | ATGGAATTAGAGAAGTACGCTAAAT | 25 | 3 |
|  |  |  |  |  |  | t1537262 | TAGAGAAGTACGCTAAATTCCT | 22 | 1 |
| ^a^ miRNA candidate position in precursor stem arm; ^b^ length of mature miRNA candidate sequence. The most cloned sequence in a candidate miRNA family are shown in red and star strand (*) in pink, respectively. | | | | | | | | | |
