# Supplementary material for: Identification and Characterization of Small RNAs in the Hyperthermophilic Archaeon Sulfolobus solfataricus
Source: PLoS One. 2012 Apr 13;7(4):e35306. doi: 10.1371/journal.pone.0035306 (PMC3325985; doi:10.1371/journal.pone.0035306)
Supplement: Table S2 — Predicted miRNA candidates targets. (DOC) [file pone.0035306.s004.doc]

Supplemental Table S2 Predicted miRNA candidates targets

| **miRNA candidate ID** | **miRNA candidate** | **Predicted Target** | **Predicted Target category** | **Alignment** |
| --- | --- | --- | --- | --- |
| t1027935 | 3 | Sso0679 (trzA) N-ethylammeline chlorohydrolase, putative | Cellular process | miRNA: 1 ATTCTCTTCTTCTCTTGT 18  |||||||||||||||| |  Sbjct: 1204 ATTCTCTTCTTCTCTTAT 1187 |
|  |  | Sso3145 (acd-7) Acyl-CoA dehydrogenase (EC: 1.3.99.-) | Lipid | miRNA: 1 ATT-CTCTTCTTC-TCTTGTG 19  \|\|\| \|\|\|\|\|\|\|\|\| \|\|\|\| \|\|  Sbjct: 34 ATTTCTCTTCTTCATCTTTTG 14 |
|  |  | Sso0605 Zn finger protein, hypothetical | Uncategotized | miRNA: 1 ATTCTCTTCTTCTCTTGT 18  \|\|\|\|\| \|\|\|\|\|\|\|\|\|\| \|  Sbjct: 163 ATTCT-TTCTTCTCTT-T 148 |
|  |  | Sso1095 Aconitate hydratase (EC: 4.2.1.3) | Energy metabolism | miRNA: 2 TTCTCTTCTTCTCTTGTG 19  \|\| \|\|\|\|\|\|\|\|\|\|\|\| \|\|  Sbjct: 1152 TT-TCTTCTTCTCTT-TG 1137 |
|  |  | Sso2950 Hypothetical protein | Hypothetical protein | miRNA: 2 TTCTCTTCTTCTC-TTGT 18  \|\|\|\|\|\|\|\|\|\|\|\|\| \|\| \|  Sbjct: 360 TTCTCTTCTTCTCCTTAT 343 |
|  |  | Sso0950 Hypothetical protein | Hypothetical protein | miRNA: 1 ATTCTCTTCTTCTCTTGT 18  \|\|\|\| \|\|\|\|\|\|\|\|\|\| \|\|  Sbjct: 803 ATTCCCTTCTTCTCTAGT 786 |
|  |  | Sso0871 (argF) Ornithine carbamoyltransferase (EC: 2.1.3.3) | Amino acid | miRNA: 1 ATTCTCTTCTTCTCTT 16  \|\|\|\|\|\|\|\|\|\|\|\|\| \|\|  Sbjct: 731 ATTCTCTTCTTCTGTT 716 |
|  |  | Sso0699 (rpl18AB) LSU ribosomal protein L18AB | Translation | miRNA: 2 TTCTCTTCTTCTCT-TGTG 19  \|\|\|\|\|\|\|\|\|\|\|\| \| \|\| \|  Sbjct: 57 TTCTCTTCTTCT-TCTG-G 41 |
|  |  | Sso2738 (abfD-2) 4-hydroxybutyryl-CoA dehydratase (EC: 4.2.1.-) | Amino acid | miRNA: 1 ATTCTCTTCTTCTCTTGTG 19  \|\|\|\|\|\|\|\|\|\| \|\| \|\| \|\|  Sbjct: 1442 ATTCTCTTCT-CTATT-TG 1426 |
| t0422506 | 3* | Sso1024 NADH-Ubiquinone/plastoquinone related | Energy metabolism | miRNA: 1 CAAGAAGGAGGAGTTAAT 18  |||||||||||| ||| |  Sbjct: 910 CAAGAAGGAGGA-TTA-T 895 |
|  |  | Sso0257 (cdc6-1) Cell division control 6/orc1 protein homolog | Replication/repair | miRNA: 2 AAGAAGGAGGAGTTAATA 19  \|\| \|\|\|\|\| \|\|\|\|\|\|\|\|\|  Sbjct: 891 AA-AAGGAAGAGTTAATA 875 |
| t1050819 | 14* | Sso1005 Conserved hypothetical protein | Hypothetical protein | miRNA: 1 CTTCGTCTA-TCCTAGCC 17  ||||||||| ||||| ||  Sbjct: 400 CTTCGTCTAATCCTA-CC 384 |
|  |  | Sso2505 Sugar transport protein | Transport | miRNA: 1 CTTCGTCTATCCTAGCC-T 18  \|\|\|\|\|\| \|\|\|\|\|\| \|\|\| \|  Sbjct: 688 CTTCGTTTATCCT-GCCCT 671 |
|  |  | Sso0928 Conserved hypothetical protein | Hypothetical protein | miRNA: 2 TTCGTCTATCCTAGCCT 18  \|\|\| \|\|\|\|\|\|\|\|\| \|\|\|  Sbjct: 1146 TTCCTCTATCCTATCCT 1130 |
|  |  | Sso1999 ATP-dependent RNA helicase homolog | Helicases | miRNA: 1 CTTCGTCTATCCTAGCCT 18  \|\|\| \|\|\|\|\|\|\|\|\| \|\|\|  Sbjct: 910 CTTTATCTATCCTATCCT 893 |
|  |  | Sso2462 DNA helicase related protein | Helicases | miRNA: 1 CTTCGTCTATCCTAGCCT 18  \|\|\|\| \|\|\|\|\|\|\| \| \|\|\|  Sbjct: 1783 CTTCATCTATCCAATCCT 1766 |
| t0009876 | 19 | Sso0878 (thrC-2) Threonine synthase (EC: 4.2.99.2) | Amino acid | miRNA: 1 AGCGTAAACGGCTGCAGATGCTG 23  ||| ||| | |||||||| ||||  Sbjct: 387 AGCATAAGCAGCTGCAGAAGCTG 365 |
| t0083352 | 20 | Sso1768 Hypothetical protein | Hypothetical protein | miRNA: 1 ATGGAATTAGAGAAGTACGCT 21  || ||||||| |||| | |||  Sbjct: 343 ATAGAATTAGGGAAG-ATGCT 324 |

^i^ ID numbers in red are targets that single PCR products are obtained by 5’ RACE assays. * represents star strand of relate miRNA candidate.
